# Notfallversorgung als risikoreicher Arbeitsplatz – Maßnahmen zum Umgang mit Gewalt gegen Beschäftigte

**DOI:** 10.1007/s00063-022-00960-2

**Published:** 2022-09-15

**Authors:** Vanessa Beringer, Tanja Wirth, Lena Kazmierczak, Sonja Reißmann, Wilfried Schnieder, Hans-Werner Kottkamp, Greta Ullrich, Albert Nienhaus, Volker Harth, Anja Schablon, Stefanie Mache

**Affiliations:** 1 Zentralinstitut für Arbeitsmedizin und Maritime Medizin (ZfAM), Universitätsklinikum Hamburg-Eppendorf (UKE), Hamburg, Deutschland; 2 Fakultät für Erziehungswissenschaft, Universität Hamburg, Hamburg, Deutschland; 3 Institut für Psychologie, Universität Jena, Jena, Deutschland; 4 Zentrale Notaufnahme, Klinikum Herford, Herford, Deutschland; 5 Zentrale Notaufnahme, Evangelisches Klinikum Bethel (EvKB), Bielefeld, Deutschland; 6 Zentrale Notaufnahme, Paracelsus-Klinik Henstedt-Ulzburg, Henstedt-Ulzburg, Deutschland; 7 Competenzzentrum Epidemiologie und Versorgungsforschung bei Pflegeberufen (CVcare), Institut für Versorgungsforschung in der Dermatologie und bei Pflegeberufen (IVDP), Universitätsklinikum Hamburg-Eppendorf (UKE), Hamburg, Deutschland; 8 Abteilung Arbeitsmedizin, Gefahrstoffe und Gesundheitswissenschaften (AGG), Berufsgenossenschaft für Gesundheitsdienst und Wohlfahrtspflege (BGW), Hamburg, Deutschland

**Keywords:** Gewaltprävention, Notaufnahme, Arbeitssicherheit, Aggression, Deeskalation, Violence prevention, Emergency department, Occupational safety, Aggression, Deescalation

## Abstract

**Hintergrund:**

Viele Mitarbeitende in der Notfallversorgung erleben verbale und körperliche Gewalt, die von Patient:innen oder Begleitpersonen ausgeht. Ziel der vorliegenden Arbeit ist es, Erkenntnisse darüber zu gewinnen, welche Maßnahmen zum Umgang mit Gewalt in der Notfallversorgung verfügbar sind und wie die Beschäftigten diese subjektiv bewerten.

**Methodik:**

Eine deutschlandweite Querschnittserhebung wurde 2020 mittels eines Online-Fragebogens durchgeführt. Der Fragebogen enthielt Items zu Maßnahmen zur Gewaltprävention, -intervention und -nachbereitung. Quantitative Daten wurden deskriptiv, Freitextangaben gemäß der qualitativen Inhaltsanalyse nach Mayring ausgewertet.

**Ergebnisse:**

An der Erhebung nahmen 349 Personen, davon 115 Leitungskräfte, teil. Die Verfügbarkeit von Sicherheitspersonal und eine standardisierte Meldung von Vorfällen wurden als wichtige Maßnahmen zum Umgang mit Gewalt erachtet. Von den Leitungskräften gaben 67 % an, keinen Sicherheitsdienst zu haben, während 56 % berichteten, dass gemeldete Gewaltereignisse nicht strukturiert aufgearbeitet werden. Eine hohe Arbeitsbelastung in der Notfallversorgung kann die Umsetzung von Maßnahmen behindern. Insgesamt wurde die Forderung nach verstärkter Unterstützung durch Vorgesetzte und die Klinikleitung deutlich.

**Schlussfolgerung:**

Es zeigt sich, dass Beschäftigte bestimmte Maßnahmen als wirksam erachten, diese oft aber nicht konsequent umgesetzt werden. Es bedarf einer strukturierten Meldung von Gewaltvorfällen gegen Beschäftigte, um die Prävalenz realitätsnah abbilden zu können. Neben der Aufstockung des (Pflege‑)Personals kann der eingeschränkte Zutritt für Begleitpersonen zu einer Reduktion des Konfliktpotenzials führen.

## Abstract (en)

**Background:**

Many employees in the field of emergency care experience verbal and physical violence caused by patients or visitors. The aim of this project is to gain insights into which measures are available to deal with violence in emergency care and how employees subjectively evaluate them.

**Methods:**

A nationwide cross-sectional online survey was conducted in 2020. The questionnaire contained items on violence prevention, intervention, and follow-up measures. Quantitative data were analysed descriptively and free text data according to Mayring’s qualitative content analysis.

**Results:**

A total of 349 respondents, including 115 supervisors participated in the survey. The availability of security staff and standardised reporting of incidents were considered important measures for dealing with violence. Of the managers, 67% reported not having a security service, while 56% claimed that reported incidents of violence were not dealt with in a structured way. A high workload in emergency care can impede the implementation of measures. Overall, the demand for increased support by supervisors and the hospital management became clear.

**Conclusion:**

It is evident that employees consider certain measures to be effective; however, these measures are often not consistently implemented. There is a need for structured reporting of violent incidents against employees to facilitate a realistic recording of the prevalence. In addition to increasing the (nursing) staff, restricting access for visitors can decrease the conflict potential.

In Deutschland gehört das Gesundheitswesen zu den Wirtschaftszweigen mit den meisten Meldungen von Gewaltunfällen [6]. Gewalt gegen Beschäftigte im Gesundheitswesen geht überwiegend von Patient:innen oder deren Angehörigen aus [24]. In der Notfallversorgung werden neben den Beschäftigten des Rettungsdiensts [11] insbesondere Mitarbeitende in Zentralen Notaufnahmen (ZNA) Opfer verbaler und körperlicher Gewalt [7, 16]. Die International Labour Organization [12] (S. 4) definiert Gewalt am Arbeitsplatz als *„jede Handlung, Begebenheit oder von angemessenem Benehmen abweichendes Verhalten, wodurch eine Person im Verlauf oder in direkter Folge ihrer Arbeit schwer beleidigt, bedroht, verletzt oder verwundet wird.“*

Bei einer Befragung in den notfallmedizinischen Bereichen der Charité Berlin gaben 93 % des pflegerischen und 98 % des ärztlichen Personals an, innerhalb der letzten sechs Monate Opfer von verbaler Gewalt geworden zu sein. Von körperlicher Gewalt waren 46 % des pflegerischen und 22 % des ärztlichen Personals betroffen [14]. Vielerorts findet keine standardmäßige Erfassung von Gewaltvorfällen und nur eine selektive Meldung derer statt [2, 20], weshalb von einer Dunkelziffer auszugehen ist.

Studien zeigen, dass nachts die Wahrscheinlichkeit von Übergriffen am höchsten ist [11, 25]. Weitere Risikofaktoren sind Alkohol- oder Drogenkonsum, psychiatrische und neurologische (z. B. Schizophrenie, Demenz) sowie schmerzhafte Erkrankungen der Patient:innen [4, 5, 11, 19, 22, 25]. Auch das hohe Patient:innenaufkommen in ZNA und damit verbundene lange Wartezeiten tragen zu Gewaltvorfällen bei [5, 11].

Neben körperlichen Verletzungen kann das Erleben von Gewalt schwerwiegende, langfristige Folgen für die psychische Gesundheit der Betroffenen (z. B. Burnout) haben [7, 19]. Daraus ergeben sich negative Konsequenzen, insbesondere vermehrte Kosten für das Krankenhaus durch erhöhte Arbeitsausfälle, verminderte Produktivität und geringere Versorgungsqualität [7, 15]. Ein vorbeugender und lösungsorientierter Umgang mit Gewalt am Arbeitsplatz in der Notfallversorgung ist folglich von großer Bedeutung. Hierbei sind drei Formen von Maßnahmen essenziell: Präventionsmaßnahmen zielen darauf ab, potenziell aggressive Personen zu deeskalieren und Gewaltvorfälle im Vorfeld zu verhindern. Interventionsmaßnahmen werden angewandt, um während eines Übergriffs die Kontrolle über die aggressive(n) Person(en) zu erlangen und damit weiteren Schaden zu vermeiden. Maßnahmen zur Nachbearbeitung dienen dazu, die Gesundheit derjenigen aufrechtzuerhalten, die der Aggression ausgesetzt waren, den Gewaltvorfall zu bewerten und angemessen darauf zu reagieren [23]. Wenn im Folgenden von *Maßnahmen zum Umgang mit Gewalt* (oder nur *Maßnahmen*) gesprochen wird, schließt dies Maßnahmen zur Gewaltprävention, -intervention und -nachbearbeitung ein. Dabei liegt der Fokus ausschließlich auf Gewalt, die von Patient:innen und/oder Begleitpersonen ausgeht.

Die zuvor beschriebenen Maßnahmen lassen sich gemäß dem TOP-Prinzip in *technische *(z. B. Alarmsysteme), *organisatorische* (z. B. angemessene Personalstärke) und *personenbezogene* Maßnahmen (z. B. Deeskalationstraining) einteilen [10, 18]. Es zeigt sich, dass Maßnahmen zum Umgang mit Gewalt noch nicht konsequent umgesetzt werden [8], obwohl sich positive Effekte nachweisen lassen: Schulungen führen zu signifikanten Verbesserungen des Selbstvertrauens und der wahrgenommenen Selbstwirksamkeit [13, 21] sowie der Fähigkeiten im Umgang mit aggressivem Verhalten [1, 9, 13]. Zudem sind dadurch weniger Gewaltvorfälle zu verzeichnen [1]. Insbesondere die Kombination verschiedener Maßnahmen kann einen effektiven und nachhaltigen Schutz ergeben [3, 10]. Jedoch ist insgesamt die Umsetzung und Wirksamkeit von Maßnahmen in Deutschland [11, 22] und auch international [27] bislang nur unzureichend erforscht. Die vorliegende Arbeit untersucht die Verfügbarkeit und den wahrgenommenen Erfolg bzw. Misserfolg von Maßnahmen zum Umgang mit Gewalt aus der Sicht der Beschäftigten.

## Studienziel und Fragestellungen

Ziel der Arbeit ist es, Erkenntnisse aus Sicht der Beschäftigten zur Verfügbarkeit von Maßnahmen zum Umgang mit Gewalt, zu deren subjektiven Bewertung der Wirksamkeit sowie zu Faktoren, die eine Umsetzung behindern, zu gewinnen. Daraus ergeben sich folgende Forschungsfragen:

1. Welche Maßnahmen zum Umgang mit Gewalt stehen den Beschäftigten in der Notfallversorgung zur Verfügung und inwiefern werden diese genutzt?
2. Welche Maßnahmen zum Umgang mit Gewalt erleben die Beschäftigten als wirksam?
3. Welche Maßnahmen zum Umgang mit Gewalt sind aus Sicht der Beschäftigten weniger wirksam und warum? Welche Schwierigkeiten treten bei der Umsetzung der Maßnahmen auf?
4. Welche Verbesserungsansätze sehen Beschäftigte im Umgang mit Gewalt und Aggression?

## Methodik

### Studiendesign und Rekrutierung

Die deutschlandweite quantitative Querschnittserhebung in Form einer Online-Befragung fand im Erhebungszeitraum September bis Dezember 2020 statt. Voraussetzung für die Teilnahme war eine hauptamtliche Beschäftigung mit direktem Patient:innenkontakt in ZNA von Krankenhäusern oder in Rettungs- und Notarztdiensten. Eingeschlossen wurde ärztliches Personal, das Pflege- und Rettungsdienstpersonal sowie nichtmedizinisches Personal. Die Rekrutierung von Teilnehmenden erfolgte über den E‑Mail-Verteiler der Deutschen Gesellschaft für Interdisziplinäre Notfall- und Akutmedizin (DGINA), wodurch ca. 1000 Mitglieder angeschrieben wurden. Sie erhielten Informationen zum Studienziel, zum Ablauf, zu den Teilnahmebedingungen, zum Datenmanagement sowie den Link zur Teilnahme. Gemäß dem Schneeballprinzip wurden die Kontaktierten gebeten, die Einladung zur Teilnahme auch an andere Kolleg:innen weiterzuleiten.

### Erhebungsinstrument

Für die Erhebung wurde ein Fragebogeninstrument mit nichtstandardisierten Items und validierten Skalen verwendet. Der Fragebogen bestand überwiegend aus geschlossenen Fragen mit vorgegebenen Antwortkategorien oder mehrstufigen Skalen. Drei Items beinhalteten ein offenes Antwortformat. Alle Befragten (Beschäftigte und Leitungskräfte) wurden neben den soziodemografischen Daten nach der Verfügbarkeit und Anwendung von Maßnahmen zum Umgang mit Gewalt sowie nach dem Meldeprozess von Gewaltvorfällen gefragt. Zudem konnten sie in einem Freitextfeld Vorschläge zur Verbesserung der Arbeitssituation im Hinblick auf die erlebte Gewalt angeben. Leitungskräfte erhielten zusätzliche Items, mit denen die Umsetzung von Maßnahmen, die Dokumentation, Meldung und Nachbereitung von Vorfällen sowie die Haltung der Krankenhausdirektion erhoben wurde. Außerdem konnten die Leitungskräfte in zwei Freitextfeldern angeben, welche Maßnahmen aus ihrer Sicht besonders erfolgreich sind und welche Gründe sie für die Unwirksamkeit von Maßnahmen sehen.

### Datenauswertung

Bei den geschlossenen Fragen erfolgte eine deskriptive Analyse der Häufigkeiten mithilfe der Software IBM® SPSS® Statistics (Version 26; IBM, Armonk, NY, USA). Die Antworten auf die drei offen gestellten Fragen wurden durch Freitextfelder erfasst. In Form einer qualitativen Exploration wurden die Antworten mithilfe der Software MAXQDA (Version 12.3.9; VERBI GmbH, Berlin, Deutschland) nach Mayrings Prinzip der qualitativen Inhaltsanalyse [17] codiert und analysiert. Soweit möglich wurde zur Einordnung der Maßnahmen das zuvor beschriebene TOP-Prinzip verwendet, um diese entsprechend der Verhaltens- und Verhältnisprävention übersichtlich darzustellen.

## Ergebnisse

Die Studienpopulation bestand aus 349 Personen, von denen 115 (33 %) Leitungskräfte waren (20 % pflegerische und 13 % ärztliche Leitung). Von den Befragten waren 86 % in der ZNA eines Krankenhauses und 12 % im Rettungs- und Notarztdienst beschäftigt. Rund 24 % aller Teilnehmenden übten eine ärztliche und 60 % eine pflegerische Tätigkeit aus, während 7 % als Notfallsanitäter:in bzw. Rettungsassistent:in beschäftigt waren. In Tab. 1 wird die Stichprobe detailliert aufgeschlüsselt.

**Table 1:** 

	n	%^a^
*Tätigkeitsbereich*		
Krankenhaus, Zentrale Notaufnahme	300	86,0
Rettungs‑/Notarztdienst	41	11,7
Beides	6	1,7
Praxis	1	0,3
Keine Angabe	1	0,3
*Berufliche Qualifikation*		
Ärzt:in	85	24,4
Pflegekraft	208	59,6
Notfallsanitäter:in/Rettungsassistent:in	25	7,2
Anderer Beruf	31	8,9
*Berufserfahrung*		
0–5 Jahre	102	29,2
6–10 Jahre	86	24,6
11–15 Jahre	58	16,6
> als 15 Jahre	103	29,5
*Träger der Einrichtung*		
Privatgewerblich	63	18,1
Öffentlich	183	52,4
Frei	102	29,2
Keine Angabe	1	0,3
*Arbeitszeiten*		
Schichtdienst	302	86,5
Kein Schichtdienst	47	13,5
*Geschlecht*		
Weiblich	202	57,9
Männlich	146	41,8
Divers	0	0,0
Keine Angabe	1	0,3

^a^Aufgrund von Rundungen ergibt die Summe der Prozentangaben nicht immer 100

### Verfügbarkeit und Anwendung von Maßnahmen aus Sicht aller Befragten

Mit Blick auf die technischen Maßnahmen zeigt sich, dass 31 % der 349 Befragten *maschinelle Notfallsysteme* in der Einrichtung zur Verfügung haben. Von 115 Leitungskräften gaben 39 % an, einen Alarm nutzen zu können, der aktiv ausgelöst wird und durch den eine bestimmte Person(engruppe) verständigt wird. Von den Leitungskräften sagten 11 % aus, in Gefahrensituationen einen Schrillalarm verwenden zu können. Der überwiegende Teil der Leitungskräfte (50 %) gab an, keine Alarmsysteme in der ZNA zu haben.

**Figure 1:**
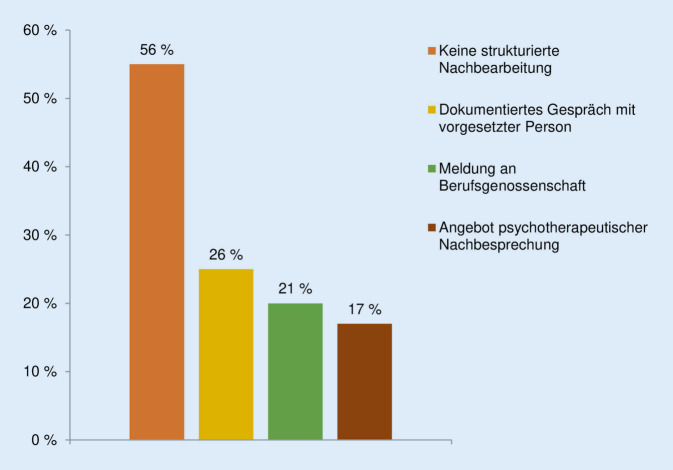

Unter die organisatorischen Maßnahmen fällt das *Sicherheitspersonal*: 32 % der 115 Leitungskräfte sagten aus, dieses in der ZNA zur Verfügung zu haben, während 67 % dies nicht bestätigen konnten. Rund 43 % derjenigen, die über Sicherheitspersonal verfügten (*n* = 37), gaben an, dass dieses 365 Tage im Jahr nachts verfügbar ist. Die Anwesenheit des Sicherheitsdiensts an 365 Tagen rund um die Uhr bestätigten 30 %, während 5 % aussagten, dass dieser nur an Wochenenden und Feiertagen vor Ort ist. Von allen 349 Befragten bestätigten 27 %, dass es in ihrer Einrichtung *Handlungsanleitungen* zum Umgang mit aggressiven Personen gibt. Die *Meldung gewalttätiger Übergriffe* im Nachgang bestätigten 35 % der 349 Befragten; 50 % melden nach eigenen Angaben lediglich schwerwiegende Übergriffe und 13 % sagten aus, keine Gewaltvorfälle zu melden. Beschäftigte, die angaben, Übergriffe zu melden (*n* = 297), richten die Meldung an Vorgesetzte (83 %), D-Ärzt:innen (53 %), die BG (18 %) oder an andere Stellen (14 %) wie z. B. Polizei oder Qualitätsmanagement. Wenn keine Übergriffe gemeldet werden, kann dies daran liegen, dass keine strukturierte Erfassung von Gewaltereignissen existiert, so wie es 43 % der 115 Leitungskräfte angaben. Etwa 34 % der Leitungskräfte sagten aus, Vorfälle schriftlich und individuell formuliert festzuhalten, während 20 % einen *standardisierten Gewalterfassungsbogen* nutzen können. Bei 29 % der 349 Befragten werden *Nachsorgegespräche* nach belastenden Ereignissen und bei 24 % *Fallbesprechungen* oder *Supervisionen* angeboten. Welche Angebote eine strukturierte Nachbearbeitung gemeldeter Gewaltereignisse nach Angaben der Leitungskräfte noch umfasst, ist in Abb. 1 dargestellt.

Mit Blick auf das *Deeskalationstraining*, das unter die personenbezogenen Maßnahmen fällt, bestätigten 58 % der 349 Befragten, dass dieses angeboten wird, während es 30 % verneinten und 12 % nicht wussten. Von jenen Befragten, bei denen dieses Angebot bestand (*n* = 201), gaben 68 % an, daran teilgenommen zu haben.

Darüber hinaus sagten 24 % der 349 Befragten aus, dass in ihrer Einrichtung *keine Angebote* zum Umgang mit Gewalt zur Verfügung stehen. In Bezug auf die Krankenhausdirektion gaben 39 % der 115 Leitungskräfte an, dass diese sich *aktiv gegen Gewalt im Krankenhaus positioniert habe*, während 58 % dies nicht bestätigten und 3 % keine Angabe machten.

### Als erfolgreich wahrgenommene Maßnahmen aus Sicht der Leitungskräfte

Von 115 Leitungskräften nahmen 31 % die in ihrer Einrichtung etablierten Maßnahmen zum Umgang mit Gewalt als erfolgreich wahr. Davon gaben 47 % (*n* = 17) Beispiele und begründeten ihre Antwort. In den Antworten kristallisierten sich elf Maßnahmen heraus, die als besonders wirksam erachtet wurden. In Bezug auf technische Maßnahmen waren dies *Aushänge* in der ZNA zur Aufklärung und Information. Hierdurch konnten Patient:innen und Begleitpersonen über Wartezeiten oder das Triage-System informiert werden, wodurch Transparenz geschaffen wurde.

Mit Blick auf organisatorische Maßnahmen wurde die Präsenz von *Sicherheitspersonal* (vor allem in den Abend- und Nachtstunden) am häufigsten genannt. Auch die Kooperation mit *Fachkräften der Psychiatrie*, die Unterstützung durch *Polizist:innen* sowie das Vorhandensein eines *Beauftragten für Konzernsicherheit* wurden als wirksam erachtet. Zu den genannten Maßnahmen, die nach Gewaltvorfällen Anwendung fanden, zählten die *Rückmeldung von Vorfällen* an die BG sowie *Konsequenzen für Täter:innen* (z. B. Hausverbot).

Unter personenbezogene Maßnahmen fallen Angebote wie *Deeskalationstrainings* oder *Fortbildungen*. Hier sollten alle Mitarbeitenden aus Sicht der Befragten einerseits lernen, adäquat mit verbaler und körperlicher Gewalt umzugehen, und andererseits, Gewaltsituationen gar nicht erst entstehen zu lassen. Dazu passend wurden auch das *eigene Verhalten und die persönliche Einstellung* genannt, wodurch Gewalt teilweise im Vorfeld verhindert werden konnte. Als wichtige Maßnahme im Nachhinein wurde die *systematische Aufarbeitung* in Form von Gesprächen innerhalb des Teams oder durch psychologische Betreuung angesehen.

### Gründe für die Unwirksamkeit von Maßnahmen und weitere Schwierigkeiten aus Sicht der Leitungskräfte

Rund 59 % der 115 Leitungskräfte erachteten etablierte Maßnahmen als erfolglos. 57 % (*n* = 39) davon begründeten ihre Antwort im Freitextfeld. Die am häufigsten berichtete Begründung bezieht sich auf Angebote wie *Deeskalationstrainings* oder *Schulungen*. Diese finden nach Angaben der Befragten zu selten oder außerhalb der Arbeitszeit statt, weshalb sie wenig Anklang finden. Alle thematisierten Gründe für die Unwirksamkeit vorhandener Maßnahmen sind in Tab. 2 stichpunktartig aufgeführt.

**Table 2:** 

Angebote finden zu selten oder außerhalb der Arbeitszeit statt
Mangelndes Problembewusstsein bei Patient:innen
Hohe Arbeitsbelastung, daher keine Aufarbeitung von Gewaltvorfällen
Ungeschützter Zugang zur Zentralen Notaufnahme
Intoxikierte Patient:innen
Anwesenheit vieler Begleitpersonen (bewirkt Unruhe und Unübersichtlichkeit)
Geringer Handlungsspielraum der Mitarbeitenden und Gefühl, „sich nicht wehren zu dürfen“
Unsicherheit beim Personal hinsichtlich des Umgangs mit Gewaltvorfällen
Unzureichende Besetzung der Pforte
Aufklärung ist unzulänglich und wirkungslos

Von allen Leitungskräften, die im Freitextfeld eine Antwort formulierten, gaben 33 % (*n* = 13) an, dass an ihrem Arbeitsplatz keine Maßnahmen zum Umgang mit Gewalt vorhanden sind. Andere Teilnehmende äußerten keine Gründe für die Unwirksamkeit etablierter Maßnahmen, sondern weitere Schwierigkeiten im Umgang mit Gewalt. Dazu zählten das Fehlen von *Sicherheitspersonal, Videoüberwachung* sowie einer standardisierten *Dokumentation und Meldung* der Vorfälle. Die Befragten bemängelten, dass *Deeskalationstrainings* aus Kostengründen eingestellt werden. Außerdem nahmen einige Leitungskräfte ein *mangelndes Problembewusstsein* bei der Klinikleitung in Bezug auf Gewaltvorfälle wahr. Sie stellten fest, dass der Fokus meist auf den *Patient:innen und deren Bedürfnissen* und weniger auf den Anliegen der Mitarbeitenden liegt. Das Verhalten der Mitarbeitenden, vor allem wenn dieses von Unzufriedenheit, Stress und Ungeduld geprägt ist, kann zudem unerwünschte Reaktionen von Patient:innen provozieren. Ferner wurde die *mangelnde Wertschätzung* für die geleistete Arbeit in der ZNA generell kritisiert und die Zunahme von Gewalt als *gesamtgesellschaftliches Problem* angesehen.

### Ansätze zur Verbesserung der Situation im Hinblick auf Gewalt aus Sicht aller Befragten

Von den 349 Befragten äußerten 32 % (*n* = 113) Vorschläge zur Verbesserung der Situation im Hinblick auf die erlebte Gewalt am Arbeitsplatz. Die *bauliche Struktur* in der ZNA wurde häufig thematisiert. Hier regten die Befragten an, die Räumlichkeiten zu entzerren, Rückzugsmöglichkeiten und Fluchtwege zu schaffen sowie Anmeldung, Wartebereich und Behandlungszimmer voneinander zu trennen. Außerdem wünschten sie sich vermehrte *Unterstützung durch Vorgesetzte*. Konkret gefordert wurde, dass eine Reaktion auf gemeldete Vorfälle erfolgt, dass klare Handlungsanweisungen (z. B. SOP) verfügbar sind, dass Vorgesetzte in Fallbesprechungen anwesend sind sowie dass ein Beschwerdemanagement für Beschäftigte eingerichtet und eine sichere Arbeitsumgebung gewährleistet wird. Die Befragten erachteten es als wichtig, dass verstärkt *Angebote zur Aufarbeitung von Gewaltvorfällen* (z. B. Supervision, Fallbesprechungen oder psychologische Betreuung) bereitgestellt werden. Eine tabellarische Auflistung aller Verbesserungsvorschläge findet sich in Tab. 3.

**Table 3:** 

Technische Maßnahmen	Entzerrung der baulichen Struktur in der Zentralen Notaufnahme
	Etablierung von Notfallknöpfen
	Videoüberwachung (im Eingangs- und Wartebereich)
	Patient:innenfreundlicher Wartebereich (z. B. WLAN, Getränke, TV)
Organisatorische Maßnahmen	Etablierung bzw. Verstärkung des Sicherheitsdiensts
	Mehr Unterstützung durch Vorgesetzte
	Mehr Personal (v. a. im Nachtdienst)
	Schnellere Abläufe und Vermeidung von Überfüllung
	Mehr Unterstützung durch Polizei
	Keine bzw. maximal zwei Begleitpersonen
	Strafrechtliche Verfolgung von Täter:innen
	Hausverbot für gewalttätige Patient:innen
	Informationen zu Schulungen sowie mehr verfügbare Termine
	Geschultes Personal, strukturierte Abläufe und angemessene Ausstattung für den Umgang mit intoxikierten und psychisch erkrankten Patient:innen
	Dokumentation und Meldung aller Gewaltvorfälle
	Vorbereitung auf Gewaltvorfälle bereits bei Einarbeitung
	Besetzung des Empfangs 24 Stunden
	Allgemeinmedizinische Praxis vor Ort zur Filterung von Patient:innen, die keiner Behandlung in der Zentralen Notaufnahme bedürfen
	Dezentralisierung der Notfallversorgung
Personenbezogene Maßnahmen	Regelmäßiges und verpflichtendes Deeskalationstraining
	Angebote zur Aufarbeitung von Gewaltvorfällen
	Fortbildungsangebote (z. B. Resilienztraining)
	Selbstbehauptung der Mitarbeitenden (sich gegen Übergriffe zur Wehr setzen)
	Verstärkte öffentliche Aufklärung zum Thema
	Schutzausrüstung für Mitarbeitende
	Mehr Wertschätzung für die Pflege (auch finanzieller Art)
	Selbstreflexion bei eigener Ungeduld und Aggression

## Diskussion

Diese Auswertung ermöglicht einen differenzierten Überblick hinsichtlich der Verfügbarkeit von Maßnahmen zum Umgang mit Gewalt und deren subjektive Bewertung aus Sicht der Beschäftigten. Einige Maßnahmen, wie die Präsenz von Sicherheitspersonal, das Angebot von Deeskalationstrainings oder Fortbildungen sowie eine systematische Aufarbeitung von Gewaltvorfällen, wurden von den Befragten als wirksam erachtet. Ein unzureichendes Problembewusstsein bei Patient:innen und der Klinikleitung, viele Begleitpersonen in der ZNA sowie eine hohe Arbeitsbelastung tragen dazu bei, dass Maßnahmen nicht ihre gewünschte Wirkung entfalten können. Insgesamt zeigen sich Forderungen nach mehr Personal, verstärkter Unterstützung durch Vorgesetzte sowie vermehrter öffentlicher Aufklärung zum Thema Gewalt gegen Beschäftigte in der ZNA.

### Technische Maßnahmen

Die Studienteilnehmenden sahen in der Umgestaltung der baulichen Struktur einen potenziellen Ansatz zur Verbesserung der Situation. Durch eine Entzerrung der Räume (z. B. Trennung von Anmeldung, Wartebereich und Behandlungszimmern) oder durch das Bereitstellen von Rückzugsmöglichkeiten für das Personal könnten gefährliche Situationen verhindert oder ein besserer Umgang damit erreicht werden. Eine Studie beschreibt, dass immer mehr Einrichtungen aufgrund von gewalttätigen Übergriffen architektonische Veränderungen (z. B. spezielle Räume zur Unterbringung aggressiver Patient:innen) vornehmen, um die Sicherheit des Personals zu gewährleisten [26].

Die vorliegende Erhebung zeigt, dass maschinelle Notfallsysteme wie beispielsweise Alarme, mit denen eine bestimmte Person(engruppe) benachrichtigt wird, bislang nur etwa in einem Drittel der Einrichtungen vorhanden sind. Eine andere Studie kommt mit 20 von 59 Häusern, in denen ein hausinternes Alarmsystem verfügbar ist, zu einem ähnlich geringen Ergebnis [8]. Die Maßnahme wurde von den Studienteilnehmenden jedoch als positiv bewertet, sie forderten die Etablierung eines Notfallknopfs. Darüber hinaus wurde vorgeschlagen, im Eingangs- und Wartebereich der ZNA eine Videoüberwachung zu installieren. Eine Maßnahme, die Teilnehmende auch in anderen Studien als sinnvoll erachteten [11]. Ob Alarmsysteme und Videoüberwachung tatsächlich zu einer Abnahme von Übergriffen oder zu einer effektiveren Intervention führen, sollte in künftigen Studien untersucht werden.

### Organisatorische Maßnahmen

Die Befragten erachteten die Präsenz von Sicherheitspersonal mehrheitlich als sinnvoll, gaben jedoch auch an, dass dieses nicht ausreichend verfügbar ist. Hier zeigt sich eine Diskrepanz zwischen einer von den Befragten als erfolgreich wahrgenommenen Maßnahme und deren tatsächlichen Umsetzung im Krankenhausalltag. Die Forderung einiger Befragten, einen Sicherheitsdienst zu etablieren oder ihn vor allem abends und nachts personell zu verstärken, findet sich auch in der Fachliteratur wieder [22].

Im Rahmen der Ansätze zur Verbesserung wurde ein beschränkter Zutritt für Begleitpersonen thematisiert. Durch zu viele Anwesende kann die Situation in der ZNA unruhig und unübersichtlich werden. Abgesehen von Behandlungen bei Kindern, Demenzerkrankten oder Sterbenden wurde es als sinnvoll erachtet, den Zutritt für Begleitpersonen gänzlich zu verbieten oder zumindest zu limitieren. Infolge der COVID-19-Pandemie ist dies geschehen, und möglicherweise könnte dies ein Modell für die Zukunft sein, da auch von Angehörigen eine Gefahr für aggressives Verhalten ausgeht [11, 19, 22]. Wie genau sich eine solche Beschränkung auf Beschäftigte und Patient:innen sowie auf die Häufigkeit von Gewaltsituationen auswirkt, sollte Gegenstand künftiger Forschungsprojekte sein.

Dass Gewaltvorfälle im Nachgang von den Beschäftigten teilweise nicht gemeldet werden, ist problematisch. Ursächlich dafür könnte sein, dass diese von vielen Beschäftigten als Teil des Jobs angesehen werden [20, 22]. Damit wird eine vollumfängliche Erfassung behindert, und es ist davon auszugehen, dass Daten zur Prävalenz häufig nicht die Realität abbilden [2]. Dass jedoch auf die Meldung eines Vorfalls durch Beschäftigte nicht automatisch eine strukturierte Nachbearbeitung (z. B. Gespräche oder Meldung an die Unfallversicherung) folgt, zeigen die Ergebnisse dieser Arbeit. Dabei ist im Rahmen der Nachbearbeitung gerade eine Meldung an die Unfallversicherung wichtig, damit diese, wenn notwendig, Hilfe anbieten und Maßnahmen zur Akutversorgung, Rehabilitation und Wiedereingliederung koordinieren kann [10]. Darüber hinaus äußerten Befragte den Bedarf, dass gemeldete Vorfälle ernst genommen werden und eine Reaktion darauf erfolgt – beispielsweise in Form von Fallbesprechungen oder psychologischer Betreuung. Hieraus ergibt sich die Forderung an die Klinikleitung, standardisierte Verfahren zur Aufarbeitung zu etablieren.

### Personenbezogene Maßnahmen

Bestehende Studien verdeutlichen, dass Schulungen wie Deeskalationstrainings wirksame Maßnahmen im Umgang mit Gewalt sind [1, 9, 13, 21] und auch die Befragten betonten, dass diese regelmäßig und verpflichtend für das ganze Team angeboten werden sollten. Gleichzeitig zeigt sich aber auch, dass das Deeskalationstraining von einem großen Teil der Mitarbeitenden nicht besucht wird. Dass das Training zu selten oder außerhalb der Arbeitszeit stattfindet, kristallisierte sich als Begründung dafür heraus. Möglicherweise gibt es noch weitere Barrieren, die einer Teilnahme entgegenwirken. In künftigen Forschungsansätzen sollte die Frage, wie man derartige Angebote niedrigschwellig und zielgruppengerecht konzipieren kann, beleuchtet werden.

Die Ergebnisse der vorliegenden Arbeit deuten darauf hin, dass Maßnahmen zur Gewaltprävention aus der Perspektive der Befragten nicht zufriedenstellend und konsequent umgesetzt werden – während in anderen Studien beobachtet wurde, dass bestimmte Maßnahmen positive Effekte erzielen können [27] und aus Beschäftigtensicht damit eine Verbesserung der Sicherheit einherginge [8]. Damit wird auch an dieser Stelle die Diskrepanz zwischen der subjektiven Bewertung einer Maßnahme durch die Befragten und deren Umsetzung in der Praxis deutlich. Ursächlich für die unzureichende Umsetzung könnte auch ein mangelndes Problembewusstsein sein – die Ergebnisse zeigen, dass sich die Krankenhausleitung teilweise nicht aktiv gegen Gewalt positioniert.

Beschäftigte berichteten zudem, dass auch die eigenen Verhaltensweisen und Einstellungen eine Rolle spielen und Gewaltsituationen damit teilweise bereits im Vorfeld verhindert werden können. Ein stark beanspruchtes Personal wiederum trägt zur Entstehung von gewalttätigen Vorfällen bei [11]. Durch den vor allem die Pflege betreffenden Personalmangel wird die Situation weiter verschärft [8, 22]. Es bedarf einer gesteigerten Wertschätzung für Pflegekräfte, in finanzieller Form vonseiten der Klinikleitung, aber auch politisch und gesamtgesellschaftlich.

### Stärken und Limitationen

Die Kombination aus geschlossenen und offenen Fragen ermöglicht tiefgehende Einblicke in die Gründe, weshalb bestimmte Maßnahmen als erfolgreich bzw. wirkungslos erachtet werden. Es wirkt sich limitierend aus, dass die gewonnenen Daten lediglich auf den Selbstauskünften der Teilnehmenden beruhen und damit subjektive Sichtweisen widerspiegeln. Es ist möglich, dass mehrere Personen, die an der Befragung teilnahmen, zum Zeitpunkt der Erhebung in derselben Einrichtung beschäftigt waren. Doppelnennungen von Krankenhäusern bzw. Orten konnten damit nicht ausgeschlossen werden. Aufgrund der Anonymität der Befragung ist dies nicht nachvollziehbar. Zudem kann wegen der Rekrutierungsstrategie, die die Verteilung des Befragungslinks über die DGINA sowie per Schneeballprinzip beinhaltete, keine Gesamt-Rücklaufquote errechnet werden.

## Fazit für die Praxis

- Eine strukturierte Dokumentation und Meldung von Gewaltvorfällen sind notwendig, um die tatsächliche Prävalenz zu bestimmen.
- Konsequentere Umsetzung von als wirksam erlebten Maßnahmen: Sicherheitsdienst, Deeskalationstraining, Aufarbeitung von Gewaltvorfällen.
- Auch Verhaltensweisen und Einstellungen des Personals können zur Entstehung von Gewaltvorfällen beitragen, weshalb Selbstreflexion wichtig ist.
- Neben mehr Personal (insbesondere in der Pflege) könnte auch der beschränkte Zutritt für Begleitpersonen zu einer Reduktion der Arbeitsbelastung und damit zu weniger Gewaltsituationen führen.
- Befragte fordern vermehrte Unterstützung durch direkte Vorgesetzte und die Klinikleitung, insbesondere eine Reaktion auf gemeldete Vorfälle, das Bereitstellen klarer Handlungsanweisungen (z. B. SOP) sowie im Allgemeinen die Gewährleistung einer sicheren Arbeitsumgebung.
